# Writers and Readers of DNA Methylation/Hydroxymethylation in Physiological Aging and Its Impact on Cognitive Function

**DOI:** 10.1155/2019/5982625

**Published:** 2019-07-14

**Authors:** Rodrigo F. Torres, Ricardo Kouro, Bredford Kerr

**Affiliations:** ^1^Centro de Estudios Científicos, Valdivia 5110466, Chile; ^2^Fundación Cultura Científica, Valdivia 5112119, Chile; ^3^Programa de Honor en Investigación, Facultad de Medicina, Universidad Austral de Chile, Valdivia 5110566, Chile; ^4^Centro de Biología Celular y Biomedicina-CEBICEM, Facultad de Medicina y Ciencia, Universidad San Sebastián, Santiago 7510157, Chile

## Abstract

The chromatin landscape has acquired deep attention from several fields ranging from cell biology to neurological and psychiatric diseases. The role that DNA modifications have on gene expression regulation has become apparent in several physiological processes, and numerous efforts have been performed to establish a relationship between DNA modifications and physiological conditions, such as cognitive performance and aging. DNA modifications are incorporated by specific sets of enzymes—the writers—and the modified DNA-interacting partners—the readers—are ultimately responsible for maintaining a functional epigenetic landscape. Therefore, understanding how these epigenetic mediators—writers and readers—are modulated in physiological aging will contribute to unraveling how aging-associated neuronal disturbances arise and contribute to the cognitive decline associated with this period of life. In this review, we focused on DNA modifications, writers and readers, highlighting that despite some methodological disparities, the evidence suggests a critical role for epigenetic mediators in the aging-associated neuronal dysfunction.

## 1. Introduction

Changes in gene expression allow the dynamic nature of the neuronal function. The role of gene expression regulation in directing neuronal states has been highlighted in synaptic plasticity and learning and memory processes [1]. However, its relevance has acquired attention from several biological processes relevant to neuronal function, such as energy metabolism, redox balance, unfolded protein response, neurological and psychiatric diseases, and physiological aging [2–4].

One of the mechanisms through which gene expression is controlled in a long-lasting manner is by the remodeling of the chromatin landscape induced by epigenetic changes, which are chemical modifications in histone proteins and DNA bases. Modifications of the chromatin landscape are functionally relevant, as it settles the accessibility of protein complexes to regulatory regions of promoters and thus direct gene expression. The epigenetic modifications are signatures through which environmental factors chemically modify the genome to leave a record and form, a many times permanent, genetic-molecular memory. In this regard, epigenetic modifications have emerged as a relevant mechanism underlying dynamic transcriptional regulation contributing to neuronal function [5], and several efforts have been made to establish a relationship between aging and epigenetic modifications [6, 7]. DNA modifications, such as DNA methylation and DNA hydroxymethylation, are dynamically incorporated by specific sets of enzymes or writers. DNA-modified interacting proteins, or readers, allow the translation of these modifications into functional transcriptional signals that modulate gene expression contributing to neuronal function.

Besides those changes in histone and DNA methylation/hydroxymethylation, aging-associated changes in epigenetic mediators—writers and readers—could be involved in the age-related changes in gene expression that underlie one of the most singular characteristics associated to this period of life, the cognitive decline. Moreover, the processes leading to epigenetic mark erasure could also contribute to aging-associated disturbances and should be considered. Therefore, a clear understanding of the age-associated expression dynamics of epigenetic writers and readers will contribute to unraveling the aging-associated disturbances of neuronal function. In this review, we focused on DNA methylation and DNA hydroxymethylation writers and readers to highlight our current knowledge of how physiological aging modifies the expression of epigenetic mediators and the relevance of these changes for the maintenance of cognitive abilities.

## 2. DNA Methylation and DNA Hydroxymethylation Writers

The covalent binding of a methyl group to carbon 5 of a cytosine (5meC) is an epigenetic mark that is subjected to dynamic modifications. 5meC occurs at a higher frequency in cytosines of CpG dinucleotides; however, 5meC in non-CpG dinucleotides has acquired an increased relevance [8]. 5meC is catalyzed by DNA methyltransferases (DNMTs), a family of proteins that bind to DNA and write the epigenome. There are two classes of DNMTs: *maintenance* DNMTs and *de novo* DNMTs. Both groups share the use of S-adenosyl-L-methionine (SAM) as a methyl donor, and both groups of DNMTs are expressed in the brain [9, 10]. DNMT1 is the main *maintenance* DNMT and is proposed to maintain DNA methylation after DNA replication [11]. On the other hand, DNMT3a and DNMT3b are the main *de novo* DNMTs and incorporate a previously nonexistent 5meC to the genome. The *DNMT3a* locus gives rise to 2 protein isoforms named DNMT3a1 and a DNMT3a2. The DNMT3a2 lacks 219 aminoterminal residues in comparison to DNMT3a1, and it has been shown to be sensitive to an increase in neuronal activity [12]. In addition, non-CpG methylation accumulates in the genome through development, reaching levels similar to that of CpG in the adult brain [8]. Non-CpG accumulation is mediated by a transient recruitment of DNMT3a during early postnatal development [13, 14]. Interestingly, DNMT3a recruitment is modulated by early-life experiences, affecting non-CpG levels in later life [14], increasing the complexity of DNA methylation signal. Interestingly, despite its postmitotic nature, DNA methylation in neurons is far from being stable and is dynamically regulated by natural and artificial stimuli [15–17].

5meC can undergo an active series of chemical modifications that lead to cytosine demethylation. This pathway involves the oxidation of methylcytosine to hydroxymethylcytosine (5hmeC), 5-formylcytosine (fC), and 5-carboxylcytosine (caC) followed by excision of fC or caC mediated by thymine DNA glycosylase coupled with base excision repair [18]. The first of these chemical reactions is mediated by the ten-eleven translocator protein family (TETs). The TET family of proteins has three members in mammals (TET1-3), and all of them catalyze the modification of 5meC to 5hmeC [18]. The TET-mediated oxidation of 5meC to 5hmeC is *α*-ketoglutarate and Fe (II)-dependent reaction, generating an interesting link to cellular metabolism [19]. TET enzymes and 5hmeC have also been shown to have a pivotal participation in learning and memory processes and are dynamically regulated by environmental stimulation, suggesting a role for this epigenetic modification in activity-dependent gene expression [20–22].

## 3. DNA Methylation and DNA Hydroxymethylation Readers

5meC regulates gene expression either directly, by inhibiting the association of transcription factors to its binding site, or indirectly via a family of proteins containing a methyl-binding domain (MBD) [23]. The MBD was first characterized in the amino acid sequence of the methyl CpG binding protein-2 (MeCP2) and is shared by the proteins MBD1, MBD2, MBD3, and MBD4. These proteins, excluding MBD3, bind to 5meC and recruit several proteins associated to chromatin remodeling [24]. The relevance of MBD proteins in the central nervous system was highlighted by the association of MECP2 mutations as the major cause for Rett syndrome (RTT, MIM 312750), a devastating neurodevelopmental disorder [25]. Interestingly, restoring Mecp2 expression in postmitotic neurons recovers RTT in mice [26], and Mecp2 ablation in later age windows is incompatible with life [27], highlighting how important is the proper reading of epigenetic signals for the maintenance of postmitotic neuronal function. Mecp2 is the most abundantly expressed member of the MBD family in the brain, and it is coded by a 4-exon locus in the X chromosome, giving rise to 2 MECP2 isoforms. In addition to its MBD, MECP2 has a transcriptional repressor domain (TRD) and a carboxy-terminal domain. The MBD is necessary and sufficient for methylcytosine binding in a DNA sequence-independent manner [28], and the TRD domain has been shown to interact with HDACs among other proteins [29]. In spite that Mecp2 was initially described as a transcriptional repressor, it was latter shown that some of Mecp2 target genes are downregulated in the brain of a mouse model lacking the expression of Mecp2, suggesting that Mecp2 is also able to activate gene expression [30]. Currently, several evidences suggest that Mecp2 acts as a chromatin structure regulator, and therefore, the effect of Mecp2 mutations or posttranslational modifications will be dependent upon the chromatin context [31, 32].

The mechanism through which Mecp2 activates the expression of its target genes is still a matter of study. It has been described that Mecp2 interacts with the cAMP response element-binding protein (CREB) [30], a widely known activator of gene expression. Considering that CREB phosphorylation and CBP recruiting are induced by neuronal activity [33], it is not risky to propose this interaction as a molecular linker between environmental factors and gene expression control.

It has also been shown that Mecp2 binds 5hmeC in the central nervous system [34]; therefore, Mecp2 represents an intersectional link between DNA methylation and hydroxymethylation. The affinity of Mecp2 for 5meC vs. 5hmeC is a matter of study that will contribute to understand how epigenetic modifications relate to the tuning of gene expression. Recent evidences suggest that MBD1 has a higher affinity for 5meC than Mecp2; on the other hand, Mecp2 seems to have higher affinity for 5hmeC than MBD1 [35]. Hence, the binding of MBD1 or Mecp2 to a certain promoter region could act to switch gene expression [35]. Thus, the control of TET-mediated oxidation activity over discrete genomic regions could have profound effects on the chromatin landscape and gene regulation, highlighting the dynamism of epigenetic changes.

Interestingly, neuronal activity modulates Mecp2 phosphorylation, decreasing phosphoserine 80 [36] and increasing phosphoserine 421 [37, 38], suggesting that Mecp2 posttranscriptional modifications are relevant to synaptic plasticity and activity-dependent gene expression [39], further highlighting the role of Mecp2 in the adult brain.

As exemplified by Mecp2 phosphorylation, there is a vast range of posttranslational modifications regulating the function of Mecp2 [39]. Understanding how these posttranscriptional modifications contribute to Mecp2 functionality in aging will be crucial to understand the physiological aging-associated neuronal dysfunction.

The information regarding Mecp2 contribution to cognitive function comes mainly from studies using mouse models of RTT. For instance, a mouse model containing a truncated form of Mecp2 shows impaired synaptic plasticity and learning and memory [40]. Similarly, the Mecp2-null mice showed a disruption of synaptic plasticity and deficits in cerebellar and amygdala-based learning [41, 42]. Of relevance to aging-associated cognitive decline, it has been shown that Mecp2-null mice show an impairment of place cell function, resulting in the incapacity for maintaining contextual memory in long-time scales [43]. Moreover, by using a knockdown approach to delete the expression of Mecp2 from the hippocampus of adult mice, Gulmez Karaca et al. reported that Mecp2 is required for long-term memory formation and consolidation [44].

## 4. Writers, Readers, and Aging

The cognitive decline associated with aging is linked to a change in gene expression that drives several neuronal functions [45, 46]. As DNA methylation and hydroxymethylation are dynamically regulated and sensitive to neuronal activity, the observation that these modifications also vary across the lifespan suggested a role for DNA modifications during physiological aging [6, 47–49]. The reader is directed to specialized works and reviews to address the aging-associated genome modifications of 5meC and 5hmeC [50–54]. However, systematic studies assessing the dynamics of the expression of epigenetic writers and readers across the lifespan are lacking and the available information raises technical hitches which contribute to variability between laboratories, such as the age of “aged mice” sampling and the age of the “young” mice used as controls. However, it is remarkable that despite these differences, similar findings have been reported which are summarized in Table 1.

Although few efforts have been made to elucidate the role of epigenetic mediators in aging, the results obtained have been highly interesting and call for further attention. Based on the general consensus that global DNA methylation decreases in the aged brain [52], Oliveira et al. investigated a possible causal role for DNMTs. They observed that the expression of DNMT3a1 and DNMT3a2 is decreased in the hippocampus of 18-month-old mice. Restoring the expression of DNMT3a2 recovered the declined cognitive performance in fear conditioning and the object-place recognition test. Inversely, decreasing DNMT3a2 levels were detrimental in young 3-month-old adult mice [12]. Concordantly, decreased DNMT1 and DNMT3a expression, but not DNMT3b, was observed in postmortem human samples and in the hippocampus of 30-month-old rats [55]. DNMT1 haploinsufficiency, evaluated at 12 months of age, has also been associated with an age-dependent cognitive impairment [56]. Altogether, this evidence suggests a pivotal role for DNMT dosage in aging.

A reduction in hippocampal TET1 and TET2 expression was observed in 18-month-old mice [57]. Concordantly, TET2 expression also showed a premature and age-dependent decreased expression in the hippocampus of mice starting at 6 months of age [58]. Diminished TET2 expression was associated to a reduction in the levels of hippocampal 5hmeC and to reduced neurogenesis in the dentate gyrus. Moreover, increasing TET2 expression in 6-month-old mice restored neurogenesis to the level observed for young 3-month-old adult mice and enhanced associative fear memory [58]. This evidence suggests a critical role for this enzyme in aging-associated cognitive decline.

As was mentioned above, MECP2 binds to either 5mC or 5hmC and mutation in its coding gene is the main cause of RTT. Since RTT symptoms arise early in development, most of MECP2 literature overlooks the role for this protein in aging. However, tissue microarray analyses showed that MECP2 expression increases along aging in humans [59, 60] and similar results were observed at mRNA levels in 28-month-old mice [61]. Moreover, Mecp2 deletion in 7-10-month-old mice is incompatible with life [27] and increased Mecp2 protein levels have been shown to be detrimental, leading to a progressive neurological disorder [62]. This evidence suggests a relevant role for Mecp2 in aging that has not been explored and remains a key piece of information to understand the role of epigenetic mediators in the aging-associated cognitive decline. Furthermore, the evidence suggests that Mecp2 is target to miRNAs [63, 64] and the MECP2 gene contains a CpG island that could be associated with its transcriptional control [65]; however, such mechanism for the regulation of Mecp2 expression in aging has not been explored.

## 5. Environmental Stimulation

Some environmental conditions attenuate or delay characteristics associated to physiological aging. Among those conditions, physical exercise (PE), caloric restriction (CR), and environmental enrichment (EE) have been shown to modulate the epigenome and contribute to improve cognitive function [66–68]. For instance, CR was shown to delay the aging-associated modifications of DNA methylation [69] and PE has widely documented effects on the epigenetic landscape (for a review, see Fernandes et al., [66]). EE is an experimental paradigm widely used to increase neural plasticity in an experience-dependent manner [68]. It was shown that EE modulates the dynamics of hippocampal DNA hydroxymethylation, with changes affecting mainly genes related to axonal guidance [21]. Moreover, it has been reported that short-term EE (3 weeks) in 21-month-old rats restores synaptic and visual plasticity, suggesting that environmental stimulation recovers, at least in part, the transcriptional changes associated to aging [70, 71]. A similar conclusion can be drawn by comparing the gene expression of 18-month-old mice with or without access to PE by a running wheel [72]. Mechanism associated with the effect of environmental stimulation over aging ranges across several neuronal processes, including diminished synaptic protein loss, metabotropic glutamatergic signaling enhancement, and increased BDNF maturation, among others [73–75]. However, it is relevant to elucidate if environmental stimulation directs changes in the expression of epigenetic writers and readers in aging.

**Figure 1:**
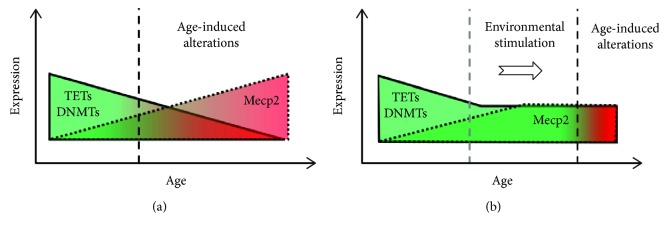
Aging-induced changes in the expression of epigenetic mediators. (a) Aging is associated with decreased DNMT1, DNMT3a1, DNMT3a2, TET1, and TET2 expression. Conversely, Mecp2 expression is increased in postmortem human samples. (b) The exposure to environmental stimulation could diminish or revert the aging-associated alterations in the expression of epigenetic mediators, as it has been observed for TET2 and physical exercise and DNMT1 activity in glucose restriction.

Although intrinsic age-associated disruption of neuronal circuits occurs during aging [76], the cognitive decline observed is also associated with a perceptual decline that could determine a reduction of neuronal stimulation due to the impoverished perceptual input, a hypothesis known as “information degradation” [77, 78]. As neuronal activity arising from environmental stimuli is required to direct the changes in DNA methylation and hydroxymethylation, the impoverished perceptual input might contribute to the aging-related changes in epigenetic mediators. Under this view, it could be hypothesized that an increase in environmental stimulation would restore, prevent, or diminish the aging-associated changes in the expression of epigenetic mediators (Figure 1). In this line, it was reported that the age-associated reduction in hippocampal TET2 expression observed in 18-month-old mice was restored to the level observed in 3-month-old mice by exposure to voluntary PE in a running wheel for 4 weeks [57]. Similarly, glucose restriction increased DNMT1 activity *in vitro*, counteracting the diminished DNMT1 expression reported for aging [79]. Interestingly, Mecp2 expression is downregulated by neuronal activity *in vitro* and it has been shown that EE reduced Mecp2 mRNA expression in 8-week-old mice [64, 80], which raises the possibility that an aging-associated reduction in neuronal activity could underlie the increase in Mecp2 expression observed in both aged humans and mice [59–61]. Supporting this notion, it was reported that in 2-month-old mice voluntary running (7 days) reduced Mecp2 expression in the cerebellum [81]. Therefore, it would be interesting to assess if EE, PE, and CR modify the level of epigenetic mediators in the brain of aged mice and the consequences at the level of gene expression and cognition. Moreover, alteration of Mecp2 protein levels could impact Mecp2 posttranslational modifications in the aged brain, contributing to aging-associated neuronal disturbances.

The aging-induced reduction in neuronal stimulation adds a layer of complexity to the interpretation of aging-related changes in gene expression. Notwithstanding, the relevance of epigenetic mediators in aging has been strikingly highlighted by some of the works reviewed, raising an urge to fulfill the voids in our understanding.

## 6. Conclusions and Prospects

As it was discussed above, physiological aging is accompanied by a cognitive performance decline associated with changes in the expression of epigenetic writers and readers that modify chromatin landscape and thus impact on gene expression. However, more evidence is required to fulfill the gaps among all these processes and take our knowledge beyond correlations between these aging-associated events. To achieve that, it is needed to unveil the mechanism underlying the changes in the expression of DNA readers and writers and the physiological factors associated with the signaling that leads to these modifications. Since the transcriptional changes that accompany aging seem to be gene-specific and do not affect the entire genome, it is critical to identify the mechanism through which DNA writers select their target regions to write on it. Then, with that knowledge, we could understand how through environmental factors these changes can be prevented or mitigated to maintain a better cognitive performance during aging, which is critical nowadays in which the life expectancy has raised.

## Figures and Tables

**Table 1:** Reported changes in DNMTs, Mecp2, and TETs expression in aging.

	Species	Age (months)	Reported change	Brain region	Reference
Dnmt1	m	18	=	Hippocampus	[12]
	m	24	↓	Frontal cortex	[82]
	r	30	↓	Hippocampus	[55]
	h	^∗^	↓	Hippocampus	[55]

Dnmt3a1	m	18	↓	Hippocampus	[12]
	r	30	↓	Hippocampus	[55]
	h	^∗^	↓	Hippocampus	[55]

Dnmt3a2	m	18	↓	Hippocampus	[12]

Dnmt3b	m	18	=	Hippocampus	[12]
	r	30	=	Hippocampus	[55]
	h	^∗^	=	Hippocampus	[55]

Mecp2	m	31	↑	Whole brain	[61]
	h	^∗^	↑	Cerebral cortex	[59, 60]

Tet1	m	18	↓	Hippocampus	[57]
	m	24	=	Frontal cortex	[82]
	m	31	↓	Whole brain	[61]

Tet2	m	18	↓	Hippocampus	[57]
	m	18	↓	Hippocampus	[58]
	m	31	↑	Whole brain	[61]

Tet3	m	18	=	Hippocampus	[58]
	m	24	=	Frontal cortex	[82]
	m	31	↓	Whole brain	[61]

Species: h: human, m: mouse, r: rat. Age is presented in months. ^∗^Human postmortem samples include varied ages.
